# Factors related to burnout in resident physicians in Japan

**DOI:** 10.5116/ijme.5caf.53ad

**Published:** 2019-07-04

**Authors:** Yoshito Nishimura, Tomoko Miyoshi, Mikako Obika, Hiroko Ogawa, Hitomi Kataoka, Fumio Otsuka

**Affiliations:** 1 Department of General Medicine, Okayama University Graduate School of Medicine, Dentistry and Pharmaceutical Sciences, Okayama, Japan

**Keywords:** Burnout, empathy, residency training, work style reform, overtime hours

## Abstract

**Objectives:**

We explore the prevalence and characteristics of burnout among Japanese resident physicians and identifies factors associated with burnout.

**Methods:**

A cross-sectional study was conducted three times between April 2017 and March 2018 at a Japanese teaching hospital. Resident physicians were invited to answer an online survey that included existing valid instruments related to burnout, depression, and empathy. Demographic, background, occupational, and socioeconomic data were also collected. Participants were prompted to report the average daily work hours and the specialty they wish to pursue.

**Results:**

Overall, 39/76 (51%), 27/76 (36%), and 21/76 (28%) resident physicians responded to surveys in April 2017, October 2017, and March 2018, respectively. The percentages of participants with burnout for surveys in April 2017, October 2017, and March 2018 were 7/39 (18%), 6/27 (22%), and 7/21 (33.3%). Emotional exhaustion (EE) was the only burnout component strongly correlated with the severity of depression (r = .615, p < .001; r = .706, p < .001; r = .601, p < .01). EE and depersonalization (DP) had no significant correlation with average daily working hours (β = .156, p = .343 for EE; β = .061, p = .711 for DP).

**Conclusions:**

The results suggest that capping working hours alone may not be effective in reducing burnout in Japanese resident physicians. Medical educators might need to consider not only working hours but also individual job quality and satisfaction to address burnout. Future studies may need to incorporate qualitative methods to explore the characteristics of burnout.

## Introduction

Burnout is a chronic psychological condition characterized by a loss of enthusiasm, feelings of physical and mental exhaustion, depersonalization, and a reduced sense of personal accomplishment.^1^ Burnout is known to be prevalent among physicians, who are constantly burdened with an enormous amount of stress and responsibility.^2^ In fact, estimates from the United States and European countries indicate that the prevalence of burnout may exceed 50%.^3^ Resident physicians are especially vulnerable to burnout when they are new to their working environment and are exposed to pressure from senior colleagues.^4^^-^^10^ In addition, physician burnout can increase the rate of medical errors, lead to suboptimal patient care, and reduce empathy. Following previous reports, researchers have been striving to pinpoint its prevalence and geographical characteristics in order to design possible countermeasures.^11^^-^^17^

The postgraduate residency training system in Japan is characterized by a national matching system and a two-year clinical training program. During this program, residents rotate through different specialties, which may require them to move to different facilities. This differs from the system in the United States, wherein residents complete three to six years of clinical training after choosing a particular specialty. It is more similar to the system in place in the United Kingdom, where all medical graduates must participate in general practitioner training as their postgraduate training. The unique qualities of Japan’s medical residency system, particularly with regard to its sources of stress, suggest that the characteristics and associated factors of burnout might differ between Japanese residents and those of other countries.

Some scholars have proposed that burnout tends to overlap with depression, although their relationship remains unclear.^5^^,^^18^^-^^21^ It was reported that 6–7% of Japanese people are depressed,^22^ which is substantially lower than the 25.2% reported among Japanese resident physicians.^23^ Taking these results together with the high burnout rates among Japanese resident physicians, we can expect a higher rate of overlap between burnout and depression in the participants as well.

Despite the urgent need to determine the prevalence of burnout in Japanese resident physicians, there are comparatively few studies on this topic, with the latest being published in 2016.^24^ It is important to update our knowledge of the prevalence and any contributing factors of burnout in resident physicians, particularly in light of the work reforms introduced by the Japanese Ministry of Health, Labour and Welfare (MHLW) in 2017. The draft of work reform plan included capping overtime work;^25^ however, it remains unclear whether mere work hour restrictions are effective in preventing burnout.

In this study, we aimed to reveal the up-to-date prevalence, characteristics, and contributing factors of burnout among Japanese resident physicians with special focus on work hours.

## Methods

### Design and study participants

We performed cross-sectional surveys between April 2017 and March 2018. Questionnaires were administered in April 2017 (Time 1), October 2017 (Time 2), and March 2018 (Time 3). Participants were the postgraduate year (PGY) 1 and 2 resident physicians at Okayama University Hospital, in Okayama, Japan. This is a university hospital with 813 beds. We distributed a self-administered online survey to all PGY 1 and 2 resident physicians (76 people) in a single Japanese university hospital through an e-mail invitation. No financial incentives were given for their participation. This study protocol was approved by the institutional review board of Okayama University Hospital. The participants’ consent was implied by their return of the questionnaires.

Of the 76 resident physicians, 39 (51%; Time 1), 27 (36%; Time 2), and 21 (28%; Time 3) responded to surveys; and the overall prevalence of burnout were 7/39 (18%), 6/27 (22%), and 7/21 (33%), respectively. In detail, the prevalence of burnout at Time 1, Time 2, and Time 3 was 4/24 (17%), 4/18 (22%), and 5/12 (42%) in PGY1 physicians and 3/15 (20%), 2/9 (22%), and 2/9 (22%) in PGY2 physicians, respectively. The demographic characteristics of the resident physicians are shown in Table 1.

### Measurements

#### The Maslach Burnout Index (MBI)

Burnout was measured using the Japanese translation of the Maslach Burnout Inventory-Human Services Survey (MBI-HSS). This instrument was validated for measuring burnout by Higashiguchi and coleagues^26^ and consists of 22 items covering three domains: emotional exhaustion (EE), depersonalization (DP), and personal accomplishment (PA). Each item is answered on a 7-point Likert scale from “never” or 0, to “daily” or 6. Because it is reported that PA was an independent variable, we defined an EE score of 27 or higher and a DP score of 10 or higher as being indicative of burnout in physicians; we, therefore, defined these individuals as “burnout positive.”

#### The Jefferson Scale of Empathy (JSE)

In this study, we used the physician version of the Jefferson Scale of Physician Empathy (JSE) to measure the level of empathy in resident physicians. The scale includes 20 items answered on a 7-point Likert-type scale (1 = strongly disagree, 7 = strongly agree). Higher scores indicate greater empathy,^27^^, ^^28^ and the highest possible score is 140.

#### Patient Health Questionnaire (PHQ-9)

We assessed the severity of depression using the 9-item Patient Health Questionnaire (PHQ-9). This is a commonly used means of screening for the presence and severity of depression. We used the Japanese translation of this scale, which was validated by Muramatsu and coleagues.^22^ The total scores of this scale range from 0 to 27; scores of 5–9 indicate mild depression, while scores of 10 or more suggest moderate or severe depression.

#### Occupational and Socioeconomic Circumstances

To assess the relationship between burnout and socioeconomic status such as living circumstances, residents were asked to provide their age, graduating school, living environment, workplace at the time of completing the questionnaires, self-reported average sleep time, and work hours in the nearest month. Because these resident physicians also engage in rotations in other university-affiliated community hospitals, they were asked about their training department and facility.

### Statistical analysis

We examined the associations among the variables using Pearson’s product moment correlation coefficient. We followed this with a linear regression analysis to further delineate the associations. The threshold for significance was p < 0.05. All statistical analyses were conducted using SPSS Statistics 22.0 (IBM Japan, Ltd., Tokyo, Japan).

**Table 1:** Demographic Characteristics of the Resident Physicians

Variable		Time 1 (n = 39)		Time 2 (n = 27)		Time 3 (n = 21)	
Mean Age (SD)		26.0 (2.2)		26.6 (2.6)		27.4 (2.7)	
		n (%)		n (%)		n (%)	
Levels of training							
	PGY1	24 (61.5)		18 (66.7)		12 (57.1)	
	PGY2	15 (38.5)		9 (33.3)		9 (42.9)	
Sex		PGY1	PGY2	PGY1	PGY2	PGY1	PGY2
	Male	9 (37.5)	10 (66.7)	3 (16.7)	6 (66.7)	1 (8.3)	7 (77.8)
	Female	15 (62.5)	5 (33.3)	15 (83.3)	3 (33.3)	11 (91.7)	2 (22.2)
Graduating school							
	OU	16 (66.7)	6 (40.0)	12 (66.7)	3 (33.3)	9 (75.0)	5 (55.6)
	Others	8 (33.3)	9 (60.0)	6 (33.3)	6 (66.7)	3 (25.0)	4 (44.4)
Living environment							
	Alone	14 (58.3)	12 (80.0)	10 (55.6)	8 (88.9)	5 (41.7)	7 (77.8)
	Alone with relatives nearby	4 (16.7)	0	4 (22.2)	0	4 (33.3)	0
	Together with someone	6 (25.0)	3 (20.0)	4 (22.2)	1 (11.1)	3 (25.0)	2 (22.2)
Marital Status							
	Single	20 (83.3)	15 (100)	15 (83.3)	9 (100)	9 (75.0)	1 (11.1)
	Married	4 (16.7)	0	3 (16.7)	0	3 (25.0)	8 (88.9)
Department of Training							
	Internal Medicine, OU	12 (50.0)	1 (6.7)	4 (22.2)	0	4 (33.3)	0
	Surgery and ED, OU	3 (12.5)	1 (6.7)	1 (5.6)	0	2 (16.7)	1 (11.1)
	Others, OU	9 (37.5)	4 (26.7)	6 (33.3)	2 (22.2)	1 (8.3)	4 (44.4)
	Internal Medicine, Away	0	2 (13.3)	3 (16.7)	5 (55.6)	1 (8.3)	1 (11.1)
	Surgery and ED, Away	0	3 (20.0)	2 (11.1)	0	3 (25.0)	1 (11.1)
	Others, Away	0	4 (26.7)	2 (11.1)	2 (22.2)	1 (8.3)	2 (22.2)
Training Facility							
	OUH	24 (100)	6 (40.0)	11 (61.1)	2 (22.2)	6 (50.0)	5 (55.6)
	Community Hospital, Okayama	0	9 (60.0)	7 (38.9)	4 (44.4)	5 (41.7)	1 (11.1)
	Community Hospital, Others	0	0	0	3 (33.3)	1 (8.3)	3 (33.3)
Preferred Specialty							
	Internal Medicine	4 (16.7)	3 (20.0)	2 (11.1)	2 (22.2)	0	3 (33.3)
	Surgery	0	3 (20.0)	0	2 (22.2)	0	4 (44.4)
	Others	8 (33.3)	4 (26.7)	7 (38.9)	3 (33.3)	6 (50.0)	2 (22.2)
	Not Determined	12 (50.0)	5 (33.3)	9 (50.0)	2 (22.2)	6 (50.0)	0
Burnout							
	Positive	4 (16.7)	3 (20.0)	4 (22.2)	2 (22.2)	5 (41.7)	2 (22.2)
	Negative	20 (83.3)	12 (80.0)	14 (77.8)	7 (77.8)	7 (58.3)	7 (78.8)
Average working hours per week [95% CI]		57.8 [54.7, 61.0]	60.9 [54.1, 67.7]	59.7 [53.7, 65.6]	61.7 [49.7, 73.6]	55.9 [48.5, 63.4]	55.7 [46.8, 64.8]
Average hours of sleep [95% CI]		6.08 [5.84, 6.32]	6.20 [5.61, 6.79]	6.06 [5.76, 6.35]	5.89 [5.32, 6.46]	6.08 [5.48, 6.69]	6.22 [5.75, 6.70]
PHQ-9 [95% CI]		4.04 [2.81, 5.28]	4.40 [1.91, 6.89]	5.67 [3.40, 7.93]	5.67 [0.64, 10.7]	5.58 [2.95, 8.22]	4.56 [0.31, 8.80]
MBI-HSS [95% CI]							
	Emotional Exhaustion (EE)	14.0 [10.3, 17.6]	16.1 [11.3, 21.0]	19.2 [13.5, 25.0]	18.6 [11.3, 25.8]	22.2 [15.5, 28.8]	15.9 [9.05, 22.7]
	Depersonalization (DP)	2.25 [0.91, 3.59]	5.00 [2.08, 7.92]	4.56 [2.77, 6.34]	5.00 [1.78, 8.22]	7.25 [3.30, 11.2]	4.67 [2.46, 6.87]
	Personal Accomplishment (PA)	25.9 [22.2, 29.5]	29.2 [24.3, 34.1]	25.6 [20.5, 30.6]	30.9 [23.6, 38.2]	28.1 [22.3, 33.9]	26.0 [18.6, 33.4]
JSE [95% CI]		116 [111, 120]	112 [102, 121]	Not assessed		Not assessed	

Time 1, April 2017; Time 2, October 2017; Time 3, March 2018 SD: standard deviation; OU: Okayama University; ED: Emergency Department; OUH: Okayama University Hospital; PHQ-9: Patient Health Questionnaire-9; MBI-HSS: Maslach Burnout Inventory-Human Services Survey; EE: Emotional Exhaustion; DP: Depersonalization; PA: Personal Accomplishment; JSE: Jefferson Scale of Empathy

## Results

### Correlation between Burnout and Depression

Table 2 shows the correlation between the burnout components (EE and DP) and depressive symptoms. Throughout the observational period, EE was significantly and positively correlated with PHQ-9 score (r = .615, p < .001 for Time 1; r = .706, p < .001 for Time 2; r = .601, p < .01 for Time 3). By contrast, a consistently weak correlation was observed between the DP and PHQ-9 scores (r = .279, p < .086 for Time 1; r = .047, p < .817 for Time 2; r = .176, p < .445 for Time 3).

**Table 2:** Correlation between Burnout Dimensions and PHQ-9

MBI-HSS	PHQ-9					
	Time 1		Time 2		Time 3	
	r	p	r	p	r	p
Emotional Exhaustion (EE)	0.615	<0.001***	0.706	<0.001***	0.601	<0.01**
Depersonalization (DP)	0.279	0.086	0.047	0.817	0.176	0.445

Time 1, April 2017; Time 2, October 2017; Time 3, March 2018

r, correlation coefficient, * p < 0.05, ** p < 0.01, *** p < 0.001

### Factors Correlated with Burnout and Depression

We performed a linear regression analysis to clarify the factors affecting the EE and DP dimensions of burnout at Time 1. Table 3 and 4 display the results of this analysis. We initially conducted a univariate analysis to detect the significant factors and followed this with a multivariate analysis featuring the statistically significant variables from the univariate analysis. Being in PGY2 (β = .318, p = .049) and hours of sleep (β = .330, p = .040) were significantly associated with DP score at baseline in the univariate analysis. In the multivariate analysis, only hours of sleep remained a significant factor and showed a positive relationship (β = .309, p = .047).

## Discussion

This is the first exploratory pilot analyses of the associated factors of burnout in Japanese resident physicians with a special focus on its relationship with work hours. Compared to previous studies conducted in Japan,^29^ we focused on finding a correlation between burnout and socioeconomic factors, including occupational circumstances, in order to inform future interventions aimed at reducing burnout. We found that approximately 18 to 33% of resident physicians experienced burnout during the study period, which was relatively smaller than the prevalence in previous reports from other countries.^1^^,^^6^^,^^10^^,^^14^^,^^30^ However, considering the fact that one third of participants may be affected by burnout, it is crucial to develop burnout prevention measures for Japanese resident physicians.

We found that residents with longer sleep hours were more prone to high DP scores. Previous studies showed that insomnia^31^^-^^33^ and lack of motivation^34^ were potential risk factors for burnout. On the other hand, higher empathy,^35^^,^^36^ and strong family support were reported to be protective factors. Contrary to our expectations, longer sleep hours had a significant impact on burnout in our study. Because of the small number of participants, the finding may not be clinically useful. Another possible explanation for this finding is that the loss of quality sleep might be a potential trigger for burnout, as previously reported in a cross-sectional study for nurses.^37^ We also found that resident physicians who had not chosen specialties at the beginning of the year were significantly more susceptible to high depersonalization at the end of the year. Because depersonalization is a psychological status characterized by callousness toward others,^38^ it is reasonable to speculate that anxiety and uncertainty about future career would influence residents’ ability to deal with interpersonal relationships, which in turn may lead to depersonalization over time. Our data did not show any correlation between empathy and burnout. It may be due to high baseline empathy scores in the study participants,^27^ and further research is needed to determine the effect of empathy on burnout in Japanese resident physicians.

There was a strong correlation between EE and depressive symptoms, which is consistent with the results of a previous study.^39^ Although there are still serious debates about whether burnout and depression are independent entities or not,^18^^, ^^21^ it is reasonable to assume that persons who possess burnout traits have a greater tendency toward depression as well. Coupled with the high prevalence of depression among Japanese,^40^ these results suggest that we need to be more cautious than other countries with the overlap of burnout and depression in resident physicians. It may be important to perform depression and burnout screening at the same time in order to better treat affected individuals, utilizing existing epidemiologic data.

Our findings suggest that work hours were not significantly correlated with burnout. According to the Japanese MHLW, the average working hours per week of resident physicians was 53.7 hours plus 13.5 hours of on-call duty.^41^ As the average total working hours per week in all three surveys was between 55.9 to 60.3 hours, we assume that our data accurately reflect this working status of Japanese resident physicians. The lack of a relationship between work hours and burnout is contrary to a previous systematic review of 19 studies from 4 Western countries.^42^ Taken together, the findings indicate that only implementing overtime hours cap may be insufficient to prevent burnout in Japanese resident physicians. This aligns with the findings of a study on Chinese neurologists, in which, besides work hours, job content and satisfaction were crucial factors related to burnout.^11^ There are a number of pros and cons of work hour restrictions. A previous study found that work hour restrictions can decrease the quality of patient care and education.^43^ In 2003, the Accreditation Council for Graduate Medical Education (ACGME) implemented a rigid work-hour restriction for resident physicians.^44^

**Table 3:** Univariate Linear Regression Analysis Predicting Burnout Components at the Baseline (n = 39)

Variable		B	SE	Emotional Exhaustion(EE)			Depersonalization(DP)		
				β	p	B	SE	β	p
Age		0.096	0.647	0.024	0.883	-0.058	0.317	-0.030	0.857
Levels of Training									
	PGY2 (vs. PGY1)	2.175	2.877	0.123	0.454	2.750	1.348	0.318	<0.05*
Sex									
	Female (vs. Male)	-0.913	2.818	-0.053	0.748	-2.068	1.341	-0.246	0.132
Graduating School									
	OU (vs. others)	-2.241	2.820	-0.130	0.432	-0.393	1.393	-0.046	0.779
Living Environment									
	Alone	ref				ref			
	Alone with relatives nearby	2.596	4.772	0.092	0.59	-0.288	2.33	-0.021	0.902
	Together with someone	-0.543	3.436	-0.027	0.875	1.295	1.678	0.13	0.445
Marital Status									
	Single (vs. married)	1.442	4.643	0.051	0.758	1.457	2.267	0.105	0.524
Department of Training									
	Internal Medicine, OU	ref				ref			
	Surgery and ED, OU	-3.288	5.022	-0.116	0.517	-3.038	2.389	-0.219	0.212
	Others, OU	-3.692	3.445	-0.203	0.292	-0.769	1.639	-0.086	0.642
	Internal Medicine, Outside	2.462	6.671	0.063	0.714	1.962	3.173	0.103	0.541
	Surgery and ED, Outside	5.128	5.625	0.159	0.369	4.462	2.676	0.283	0.105
	Others, Outside	2.962	5.022	0.105	0.559	-1.038	2.389	-0.075	0.667
Training Facility									
	Community Hospital (vs. OUH)	5.611	3.218	0.276	0.09	2.2	1.601	0.22	0.178
Preferred Specialty									
	Internal Medicine	ref				ref			
	Surgery	6.571	6.061	0.204	0.286	-2.952	3.011	-0.187	0.334
	Others	4.821	4.177	0.259	0.256	-1.536	2.075	-0.168	0.464
	Not Determined	5.454	3.944	0.315	0.176	-0.639	1.959	-0.075	0.746
Working hours		0.142	0.148	0.156	0.343	0.027	0.073	0.061	0.711
Hours of sleep		0.279	1.822	0.025	0.879	1.796	0.843	0.33	<0.05*
JSE		-0.021	0.117	-0.031	0.858	-0.055	0.061	-0.154	0.371

Time 1, April 2017, Time 2, October 2017, Time 3, March 2018 B= regression coefficient; SE=standard error of regression coefficient; β=standardized regression coefficient

While the restriction was considered to have improved patient safety and residents’ mental health issues, it also had a negative impact on residents’ graduate medical education and professional autonomy.^45^ In 2017, the ACGME relaxed these limits to allow interns to work a full 24-hour shift.^46^ In Japan, the MHLW introduced a package of work-style reform bills to promote workers’ health, which mainly involved capping overtime work hours.^25^ Although the Diet passed the law on June 29, 2018, for resident physicians, this bill cuts both ways: it may prevent overwork but may also harm their work quality and job satisfaction. The data of this study suggest that we need not to focus only on overtime hours cap, and policies need to focus also on maintaining job quality and satisfaction. Further data should be accumulated to determine how to address health problems in resident physicians.

**Table 4:** Multivariate Analysis of Factors Affecting DP Score (n = 39)

Variable		Depersonalization (DP)				
		B	SE	β	VIF	P
Levels of training				-		
	PGY2 (vs. PGY1)	2.554	1.296	0.295	1.005	0.056
Hours of sleep		1.679	0.815	0.309	1.005	<0.05*
						

VIF: Variance Inflation Factor

This study has a few limitations. First, this was a pilot exploratory study, and it was conducted in a single teaching hospital. Thus, the results may not be generalized to other populations. Second, of the resident physicians who were eligible to participate in this study, only 21 (28%) to 39 (52%) did so because of the voluntary nature of the survey. The results are therefore marred by potential selection bias, as the participants might have been more aware of the problems regarding burnout. Third, we cannot completely rule out the possibility of unobserved factors affecting the study results. Finally, because the data used for the analyses were collected from the participants via self-report, there might be some recall bias. Despite the preliminary nature of the results due to the small sample size, our study is significant in that it provides up-to-date data on burnout among Japanese resident physicians. The results highlight the future challenges in combating burnout. Although some interventions, such as yoga, holistic approaches, and stress reduction programs, have been reported as effective in preventing burnout,^47^^-^^50^ we require further insight into this area of study in relation to Japanese physicians. It is necessary to identify effective approaches to prevent burnout while taking Japanese culture into account. Future prospective observational studies are also required to validate our findings. To allow in-depth exploration into how Japanese resident physicians feel about their jobs, qualitative methods should be included in future studies.

## Conclusions

Our findings suggest that approximately one fifth to one-third of Japanese resident physicians may experience burnout. The findings also suggest that work hour capping alone may be insufficient to prevent burnout among Japanese resident physicians. The potential implication for medical education and practice is that medical educators may need to consider not only work hour but also individual job quality and satisfaction to address burnout. It is recommended that future studies include qualitative methods to facilitate an in-depth exploration of the characteristics of burnout in Japanese resident physicians.

### Conflict of Interest

The authors have no conflicts of interest to report.
